# The Twilight Sleep Propaganda: A Danger to the Race

**Published:** 1916-07-15

**Authors:** 


### The Hospital

The Workers' Newspaper of Administrative Medicine and Institutional Life, Administration, National Insurance and Health.

No. 1571, Vol. LX. SATURDAY, JULY 15, 1916. PRICE ONE PENNY.

### THE TWILIGHT SLEEP PROPAGANDA: A DANGER TO THE RACE.

At the present time a certain enterprising editor of a Sunday newspaper is opening his columns week by week to a non-medical writer who pours unceasing scorn and contempt on the medical profession for its ignorance, inhumanity, and negligence in not adopting the scopolamine-morphine method of analgesia for women in childbirth. The doctors of London come in for the lion's share of this abuse; but the hospitals of London are equally held up to ridicule because those of them that admit women for confinements do not, as a routine, adopt the method. Indeed, the suggestion is actually made that a "twilight sleep hospital" should be established in London. This campaign of abuse of the medical profession and of advertisement for a particular method of treatment has been going on in the lay press for a year or more; and inasmuch as it has attracted a good deal of attention among the public, especially among young married women, it is time that the truth about the matter should be authoritatively told. Grave charges have been and are being constantly reiterated against the medical profession, supported by dogmatic statements. The fact that those who bring these charges are not capable of judging whether their statements are true or false is not appreciated by many members of the public; so a brief statement of the whole position may be timely.

By the use of two very powerful drugs—scopolamine and morphine—used in a certain way, it is possible to produce in a patient a state of somnolence of which he retains afterwards no recollection, though he is not at the time asleep or unconscious in the ordinary sense of those words. If these drugs be administered to a woman in the early stage of labour, she retains afterwards little or no recollection of the painful uterine contractions. The patient feels them as they occur, and will express the sense of pain: she is drowsy rather than anæsthetised. To carry the matter a little further, the later stage of labour is characterised by pains much more severe than the comparatively brief and mild pains of the earlier stage. It is for the later pains that chloroform is advantageous; or, to put it in another way, it is these severe pains that anæsthesia (complete unconsciousness) annihilates and abolishes. Not only does chloroform (or ether) confer this great boon on the mother; it actually facilitates the task of the obstetrician. Although not wholly free from drawbacks, the use of chloroform in the second stage of labour is on balance advantageous from the purely scientific, as well as from the humanitarian, point of view. There are plenty of women who prefer not to have it, just as there are plenty of people who prefer to have their teeth extracted without any mitigation of the pain; but the consensus of professional opinion is in favour of this method of anæsthesia for the later stage of labour.

Now the use of chloroform, so valuable for the later stage of labour, is detrimental in the earlier stage. Scopolamine-morphine, on the other hand, is of value only for this stage, not for the later stage. Obviously, if from the obstetric standpoint there is no countervailing disadvantage, scopolamine-morphine is as much to be recommended for the early stage as chloroform is for the later. It has to be borne in mind that the degree of pain is very much slighter in the former than in the latter; but that alone is no argument for not relieving it, if this can be accomplished without any detriment. This is where the medical profession and its critics part company. The profession says that the twilight sleep method has drawbacks, one of which is so grave that it outweighs all the advantages in ordinary cases. Those who are conducting the press campaign aforesaid deny most vehemently the existence of these drawbacks: and accuse the medical profession of inhumanity, selfishness, pigheadedness, ignorance, stupidity, and other defects of character as an explanation of the difference of opinion.

We have taken some trouble to ascertain from leading obstetric specialists in London their experience and their views on twilight sleep. We note, by the way, that the great majority of expert opinion in America and in other countries, as expressed in high-class medical literature, is in accordance with that of London. Every single man and woman practitioner we have consulted has tried the method, either in hospital or in private practice or in both. Every single one says the same thing, that twilight sleep is dangerous to the babies and that it actually kills a certain number of them. A small proportion of children are killed, but their number is a quite definite one. There the question ends in a deadlock for the present: the experts say one thing; their non-medical critics deny it, and accuse them of having bungled in their method of producing twilight sleep. We are far from any belief in the infallibility of medical experts, even when they are unanimous, as in this case they seem to be. We admit the possibility of their being one and all mistaken in their observations, illogical in their inferences, incompetent in their technique. But before we accept the word of a lay critic that such is the case, we are bound to examine the statements of that critic. We find them a mass of both *suggestio falsi* and *suppressio veri*, permeated throughout by error, exaggeration, malice, and prejudice. Their appeal is to ignorance and to hysteria, not to enlightenment and reason. The editor who publishes wild words of this sort cannot shelter himself behind his inability to judge about such technical matters; it is his duty to take counsel with responsible people. If the Editor of the *Weekly Dispatch* had followed that course, we feel convinced he would never have opened his pages to a class of sensationalism so very much contrary to the public interest as the rubbish on twilight sleep which he has printed in the last few weeks.

